# Reevaluating hypercoagulability in the portal vein and the impact of transjugular intrahepatic portosystemic shunt on platelet activation in cirrhosis

**DOI:** 10.1016/j.rpth.2026.106977

**Published:** 2026-09-29

**Authors:** Viktoria Möller, Anselm Kunstein, Christian Weigel, Stephanie D. Wolf, Alexander Killer, Matthias Dille, Mihael Vucur, Kai Jannusch, Eric Tietz, Tom Luedde, Peter Minko, Johannes G. Bode, Carolin Angendohr

**Affiliations:** 1 Department of Gastroenterology, Hepatology and Infectious Diseases, Medical Faculty at Heinrich Heine University Düsseldorf, Heinrich Heine University, Düsseldorf, Germany; 2 Department of Diagnostic and Interventional Radiology, Faculty of Medicine & Dusseldorf University Hospital, Heinrich Heine University, Düsseldorf, Germany

**Keywords:** flow cytometry, hypertension, platelet activation, portal, portal vein, vascular/pathology

## Abstract

**Background:**

In liver cirrhosis, dysregulated hemostasis predisposes patients to both hemorrhagic and thrombotic complications. The portal vein (PV) is a frequent site of thrombosis, but the underlying mechanisms remain incompletely understood. Whether the PV constitutes a locally hypercoagulable environment remains controversial, as previous studies have reported conflicting findings regarding portal venous platelet activation, endothelial dysfunction, and coagulation factor alterations.

**Methods:**

We enrolled 34 patients with liver cirrhosis undergoing transjugular intrahepatic portosystemic shunt (TIPS) implantation. Platelet count, activation, and reactivity were assessed by flow cytometry before and after TIPS, as well as across multiple vascular compartments, including the PV, superior mesenteric vein, hepatic vein, and peripheral vein. In addition, we measured key coagulation factors involved in secondary hemostasis in the indicated vessels.

**Results:**

Platelet counts declined with advancing liver disease, but no changes in platelet activation or reactivity were observed. Despite elevated platelet counts in the PV, we found no evidence of increased platelet activation or reactivity. Moreover, no compartment-specific differences were detected in the selected conventional coagulation parameters assessed, except for lower fibrinogen levels in the PV compared with the hepatic vein. TIPS implantation did not lead to a short-term increase in platelet activation.

**Conclusions:**

In this cohort, we found no evidence of increased portal venous platelet activation or reactivity. Apart from lower fibrinogen levels in the PV compared with the hepatic vein, the selected conventional coagulation parameters assessed did not show consistent compartment-specific differences. These findings do not support the concept of a PV-specific hypercoagulable state as assessed by the present platelet activation and reactivity measures and selected coagulation assays. Rather, they suggest that relief of portal hypertension and reduction of venous stasis may be more relevant for thrombosis risk than changes in platelet activation alone.

## Essentials

• Cirrhosis alters hemostasis and increases risk of portal vein thrombosis.
• Platelet activation and coagulation were assessed before and after transjugular intrahepatic portosystemic shunt.
• No evidence of increased platelet activation in the portal circulation.
• Transjugular intrahepatic portosystemic shunt did not induce platelet activation; hemodynamics may drive risk.

## 1 Introduction

Cirrhosis of the liver represents the end stage of various chronic liver diseases, most commonly alcohol-associated liver disease, viral hepatitis, and metabolic dysfunction-associated steatotic liver disease [1], the latter being of increasing socioeconomic relevance [2]. The progressive decline in liver function leads to impaired synthesis of both procoagulant and anticoagulant factors, resulting in a predisposition to hemorrhagic and thrombotic complications [3]. A serious and frequent thrombotic complication is nontumoral portal vein thrombosis (PVT), which is particularly prevalent in patients with advanced liver cirrhosis (referred to as cirrhosis from now on) [[4], [5]].

The exact pathophysiological mechanisms underlying PVT development are not fully understood and remain a matter of ongoing debate [[6], [7]].

It has been suggested that the portal venous system, in particular, may be a favored site for thrombosis due to altered hemodynamics, endothelial dysfunction, and local hypercoagulable conditions [8], [9], [10], [11], [12]. Progressive destruction of the hepatic microarchitecture and the resulting hemodynamic alterations—including a marked increase in portal venous pressure—further exacerbate this hypercoagulable environment [13]. While the role of procoagulant and anticoagulant factor imbalances in cirrhosis has been extensively studied [14], platelet activation and platelet reactivity have gained increasing attention as relevant contributors to the complex hemostatic alterations in cirrhosis [15], [16]. Recent studies have linked altered platelet function, increased platelet aggregation, reticulated platelet activation, and platelet-related indices to PVT, hepatic decompensation, and adverse clinical outcomes [10], [16], [17], [18], [19], [20]. However, their assessment remains challenging, particularly in patients with cirrhosis-associated thrombocytopenia, as platelet function assays may be influenced by platelet count and assay conditions [21], [22], [23]. Moreover, the heterogeneous effects of anticoagulation, with PVT progression even during treatment [24], [25], suggest that targeting platelet activation could offer a promising therapeutic avenue for modifying PVT progression or preventing its onset [17].

In this context, the placement of a transjugular intrahepatic portosystemic shunt (TIPS)—a procedure that establishes an artificial shunt between the portal and hepatic veins to reduce portal hypertension—may further modify the occurrence of PVT. TIPS placement in combination with anticoagulation therapy, and when appropriate with lysis therapy, has been investigated as a treatment for PVT, particularly with the aim of improving hemodynamics within the portosystemic circulation [[26], [27]. However, the insertion of the stent itself has the potential to promote thrombogenesis, which could lead to TIPS occlusion or stenosis [[28], [29]].

Despite these recognized risks, the recently published European Association for the Study of the Liver TIPS guidelines [30] still do not provide standardized recommendations for postinterventional antithrombotic management, leaving therapeutic decisions largely to the discretion of the treating physician [31].

The present study aimed to investigate whether the abdominal veins of patients without preexisting PVT undergoing TIPS implantation show evidence of a local prothrombotic milieu as assessed by flow cytometry–based platelet activation markers and selected conventional parameters of secondary hemostasis. Additionally, it was assessed whether TIPS placement causes short-term platelet activation, which could necessitate antiplatelet therapy similar to that required after coronary stent implantation.

## 2 Methods

Written informed consent was provided by all participants. The study was approved by the Ethics Committee of the Medical Faculty at Heinrich Heine University Düsseldorf (study number 2023-2386).

### 2.1 Study design and patient population

This study included patients who underwent TIPS implantation at the Department of Gastroenterology and the Institute for Diagnostic and Interventional Radiology, University Hospital Düsseldorf, between January 2023 and March 2025 (*n* = 157, extended cohort). Between March and August 2024, platelet activation and platelet counts were analyzed in predefined veins in patients undergoing TIPS implantation (*n* = 34, selected cohort).

Exclusion criteria for the study were lack of informed consent or use of platelet activation inhibitors such as clopidogrel or aspirin. Due to missing samples and/or incomplete follow-up, the number of patients available for individual analyses varied. All analyses were performed using available cases, and the respective sample size is reported for each analysis.

### 2.2 Sample collection

Prior to TIPS implantation, peripheral blood samples were taken from the cubital vein as part of routine blood sampling. Further samples were taken 1 day after TIPS implantation. At each time point, blood was collected in citrate tubes for coagulation analysis and flow cytometry, and in ethylenediaminetetraacetic acid tubes for determining platelet count.

Intraprocedural blood samples were obtained from the hepatic, portal, and superior mesenteric veins (SMVs) using the catheters placed for TIPS prior to stent implantation. To avoid biased results, the first few milliliters of blood were discarded. Blood was then aspirated into 20 mL syringes and immediately transferred into citrate tubes. Flow cytometric analysis was performed immediately after sample collection.

### 2.3 Flow cytometric assessment of platelet activation

Platelet activation was assessed by flow cytometry using antibodies against activated integrin αIIbβ3 and the degranulation marker P-selectin. Platelet reactivity was assessed both at baseline and in response to specific agonists [[23], [32]].

Platelet activation was assessed by fluorescence-activated cell sorting (FACS) using plasminogen activator inhibitor 1 (fluorescein isothiocyanate-conjugated) (Becton Dickinson/Ref.: 340507) and CD62P (APC-conjugated [Fluorescein isothiocyanate]) (Becton Dickinson/Ref.: 348107) antibodies. Each flow cytometry (FACS) tube received 3 μL of PAC-1-fluorescein isothiocyanate and 3 μL of CD62PAPC antibody. For stimulation, 3 μL of 100 μM adenosine diphosphate (ADP) solution (Sigma-Aldrich, catalog number: A2754) (final concentration: 10 μM) or 3 μL of 2000 μM protease-activated receptor 4 (PAR4) agonist solution (Anaspec, catalog number: AS60218-5) (final concentration: 200 μM) was added. Unstimulated tubes served as baseline controls. A total of 21 μL of 1:10 diluted whole blood was added per tube. Samples were incubated for 15 minutes at room temperature in the dark. Reactions were stopped by adding 700 μL phosphate-buffered saline. Samples were immediately analyzed on a Becton Dickinson FACSCanto II flow cytometer.

### 2.4 Flow cytometry acquisition and gating

Platelet populations were identified based on forward and side scatter properties and previously defined by CD41 staining in preliminary experiments. A platelet gate was defined accordingly. Acquisition was set to terminate once 10,000 gated platelet events had been recorded (Supplementary Figure S3).

### 2.5 Assessment of secondary hemostasis parameters

All of the following coagulation parameters were measured in the central laboratory of the University Hospital Düsseldorf as part of routine clinical care. Analyses were performed in sodium citrate plasma using an automated coagulation analyzer (Sysmex CS-5100, Siemens Healthineers).

The following validated assays were used: Prothrombin time/international normalized ratio (PT/INR) was determined using Thromborel S (Siemens Healthineers), a lyophilized human placental thromboplastin reagent, with clot-based detection and results expressed as INR. Activated partial thromboplastin time (APTT) was measured using Dade Actin FS (Siemens Healthineers), a liquid reagent containing purified soy phosphatides and ellagic acid, with clot-based detection and results expressed in seconds. Thrombin time was assessed using Test Thrombin Reagent (Siemens Healthineers), containing lyophilized bovine thrombin (1.5 IU/mL), with clot-based detection and results expressed in seconds.

Fibrinogen concentration was quantified using Dade Thrombin Reagent (Siemens Healthineers), containing lyophilized bovine thrombin (∼100 IU/mL), according to the Clauss method, with results expressed in g/L. Antithrombin III activity was determined using Berichrom Antithrombin III (A) (Siemens Healthineers) by a chromogenic assay and expressed as a percentage of normal activity. Factor XIII activity was measured using Berichrom F XIII (Siemens Healthineers), a chromogenic assay based on nicotinamide adenine dinucleotide, reduced form, consumption monitored at 340 nm, with results expressed as a percentage of normal.

### 2.6 Data analysis

Data analysis was performed using FlowJo 7.6.1. Positive staining thresholds for PAC-1 and CD62P were defined using histograms from unstained controls. Median fluorescence intensity of PAC-1 and CD62P signals was calculated for each condition.

### 2.7 Additional data sources

Clinical parameters, including blood test results, were extracted from routine diagnostic records collected during patient care at the University Hospital Düsseldorf.

### 2.8 Statistical analysis

Statistical analyses were performed using GraphPad Prism 8.3. Group comparisons were analyzed using Friedman tests and Wilcoxon signed-rank tests for paired samples. Correlation analyses were performed using Spearman’s rank correlation. Data are presented as median and IQR, unless otherwise specified. Bar graphs with individual data points (scatter overlay) were used for visual presentation. ∗ *P* < .05; ∗∗ *P* < .01; ∗∗∗ *P* < .001.

## 3 Results

### 3.1 Platelet distribution in cirrhosis and differences across vascular compartments

It is well known that progressive cirrhosis is typically associated with a decline in platelet counts. Consistent with this, a significant reduction in platelet counts was observed in patients with Child–Pugh C-stage cirrhosis compared with those having A- and B-stage cirrhosis (Figure 1A). The inverse relationship between platelet count and the severity of cirrhosis was further supported by a correlation analysis between peripheral platelet count and the Model for End-Stage Liver Disease (MELD) score (*r* = −.3313, *P* = .0005; [Figure 1B). Notably, the MELD score correlated significantly with PAR4-induced CD62P expression, indicating an association between disease severity and platelet α-granule release as assessed by CD62P surface expression upon PAR4 stimulation (Figure 1C).

**Figure 1:**
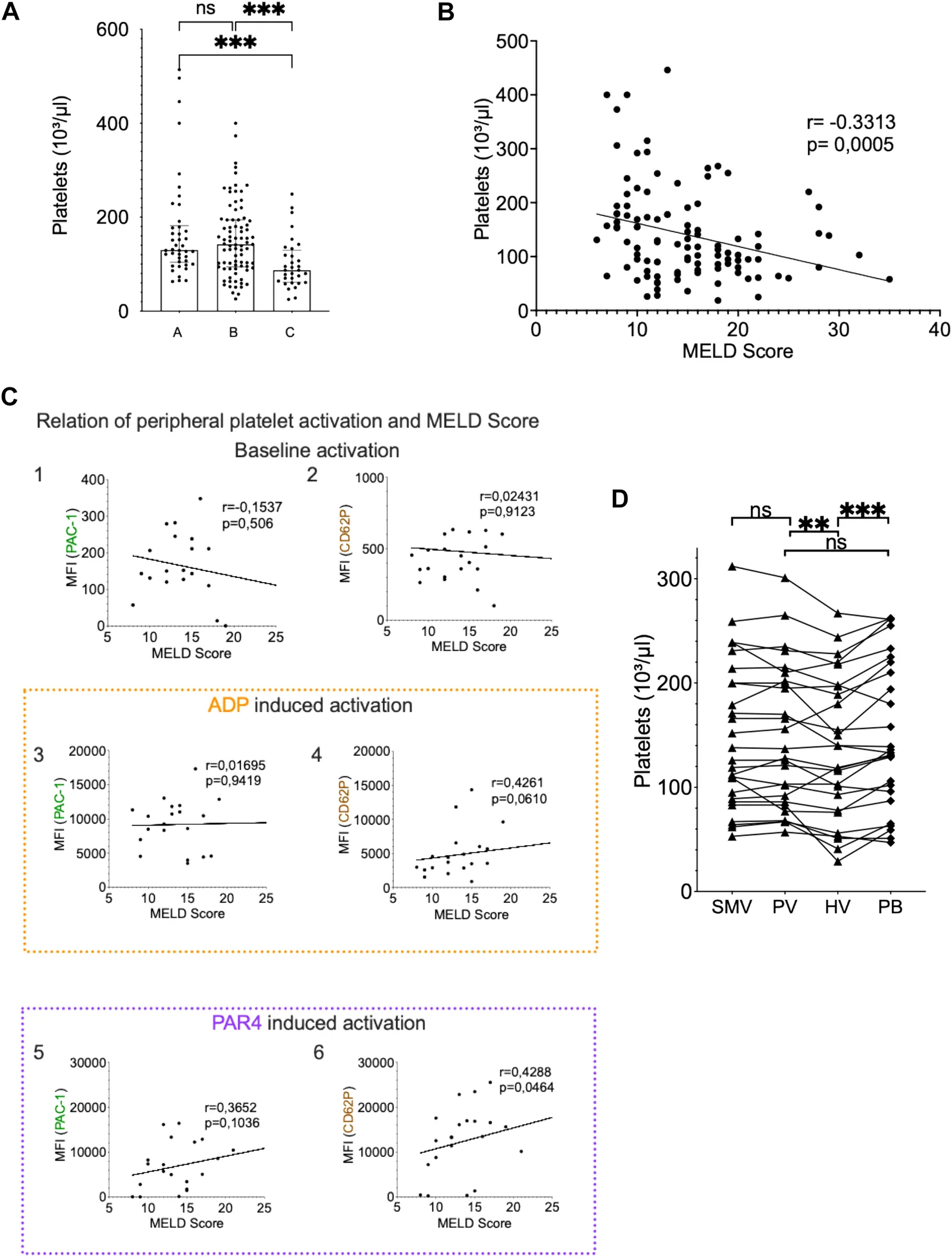
Platelet counts decrease with advancing cirrhosis and differ across vascular compartments. (A) Platelet count (10^3^/μL) in peripheral blood stratified by Child–Pugh class A, B, and C. Counts were significantly lower in class C than in A and B (*n* = 157). (B) Peripheral platelet count inversely correlated with the Model for End-Stage Liver Disease (MELD) score (*n* = 106; *r* = −0.3313, *P* = .0005). (C) Scatter plots showing the correlation between the MELD score and platelet activation assessed by PAC-1 and CD62P expression at baseline (1-2), after adenosine diphosphate (ADP) stimulation (3-4), and after PAR4 stimulation (5-6). No significant association was observed for PAC-1 expression (*n* = 19-21). (D) Platelet counts in the superior mesenteric vein (SMV), portal vein (PV), hepatic vein, and peripheral venous blood (PB). Platelet counts were significantly lower in the liver vein compared with the PV, whereas no significant differences were observed between PV, SMV, and PB (*n* = 26). Statistical analysis: Wilcoxon signed-rank test and Friedman test. HV, hepatic vein; MFI, median fluorescence intensity; PAR4, protease-activated receptor 4.

Platelet counts were then compared across distinct vascular compartments. A significant difference was detected between the liver vein and the portal vein (PV), with platelet counts significantly elevated in the PV. Conversely, platelet counts did not differ significantly among the PV, SMV, and peripheral venous blood (PB) (Figure 1D).

### 3.2 No evidence of local platelet hyperactivation in the PV

We next assessed platelet activation across vascular compartments in a selected patient cohort, whose characteristics are shown in the [Table. Platelet activation in SMV, PV, liver vein, and PB was assessed by flow cytometry using PAC-1 and CD62P staining. Median fluorescence intensity was measured under baseline conditions and following stimulation with ADP or PAR4 agonists. Baseline expression of PAC-1 and CD62P did not differ significantly across vascular compartments (Figure 2A, B). Similarly, ADP-induced (Figure 2C, D) or PAR4-induced platelet activation (Figure 2E, F) revealed no significant intercompartment differences. These findings suggest comparable levels of platelet reactivity and baseline activation in the different vascular beds investigated. Interestingly, platelet reactivity and baseline activation displayed a trend toward correlation with PV flow velocity, particularly concerning CD62P expression (Figure 3).

**Table:** Baseline characteristics of the selected cohort

Variable	Overall (*n* = 34)
Age (y), median (IQR)	59 (53-68)
Sex, *n* (%)	
Male	25 (73.5)
Female	9 (26.5)
Child–Pugh score, median (IQR)	8 (7-9)
Child–Pugh class, *n* (%)	
A	4 (11.8)
B	24 (70.6)
C	6 (17.6)
MELD score, median (IQR)	15 (12-18)
INR, median (IQR)	1.4 (1.3-1.5)
Platelet count, ×10^9^/L, median (IQR)	137.5 (96-210)
TIPS indication, *n* (%)	
Refractory ascites	24 (70.6)
Variceal bleeding	10 (29.4)
Etiology of liver cirrhosis, *n* (%)	
Alcohol-associated liver disease (ALD)	16 (47.1)
MASLD/MASH	6 (17.6)
MetALD	5 (14.7)
Cryptogenic	6 (17.6)
Other (α1-antitrypsin deficiency)	1 (2.9)

Baseline demographic and clinical characteristics of the overall study population (*n* = 34).

Values are reported as median (IQR) or *n* (%).

**Figure 2:**
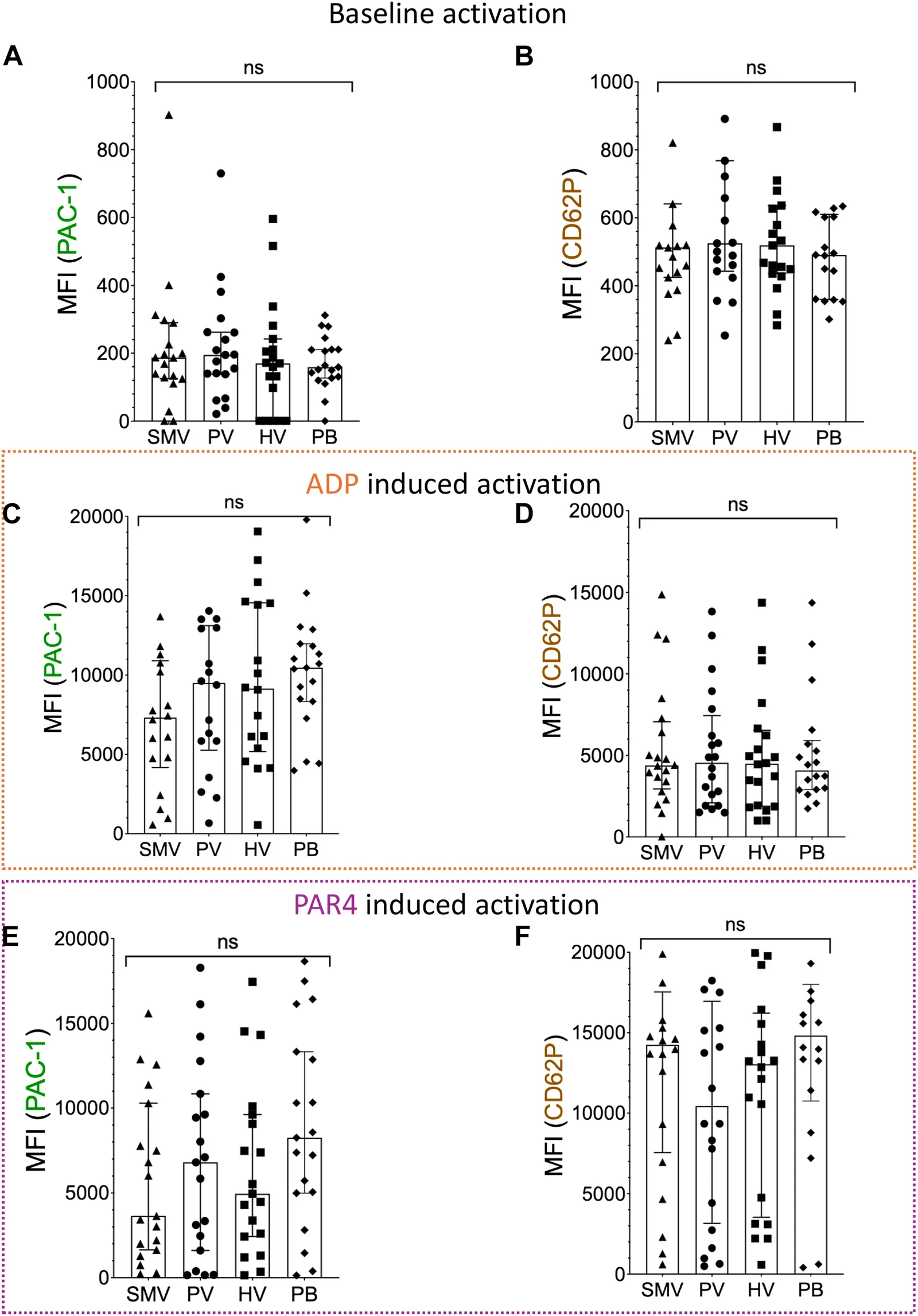
Basal activity as well as inducible activation of platelets did not significantly differ between the different compartments investigated. Platelet activation was assessed in the superior mesenteric vein (SMV), portal vein (PV), hepatic vein (HV), and peripheral venous blood (PB) using flow cytometry with PAC-1 and CD62P staining (*n* = 20). Baseline platelet activation showed no significant differences in PAC-1 (A) or CD62P (B) expression between compartments. Adenosine diphosphate (ADP)-induced platelet activation showed no significant differences in PAC-1 (C) or CD62P (D). PAR4-induced platelet activation similarly showed no significant differences in PAC-1 (E) or CD62P (F) expression. Statistical analysis: Friedman test. MFI, median fluorescence intensity; PAR4, protease-activated receptor 4.

**Figure 3:**
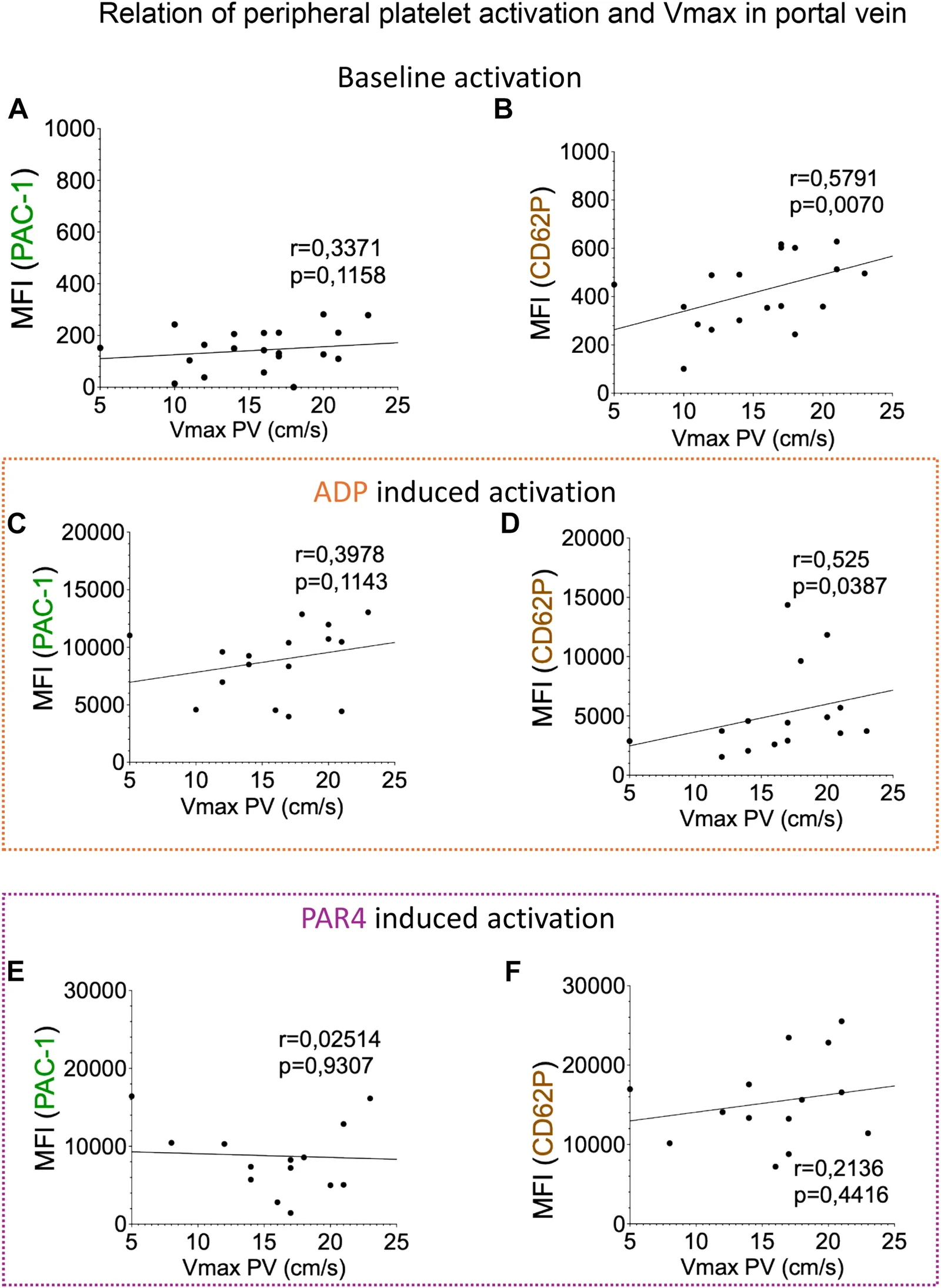
Selective increase in CD62P expression with elevated portal flow velocity before transjugular intrahepatic portosystemic shunt (TIPS). Scatter plots show the association between platelet activation and portal vein (PV) flow velocity (Vmax) prior to TIPS implantation. Platelet activation was assessed using PAC-1 and CD62P expression at baseline (A, B), after ADP stimulation (C, D), and after PAR4 stimulation (E, F). A positive correlation was observed for CD62P expression at baseline and after adenosine diphosphate (ADP) stimulation, whereas PAC-1 expression showed no significant association (*n* = 21-25). These findings suggest that platelet alpha-granule release, reflected by CD62P expression, increases with higher portal venous flow velocity, indicating a selective aspect of platelet activation in this setting. Statistical analysis: Spearman correlation; linear regression lines are shown for illustration. MFI, median fluorescence intensity; PAR4, protease-activated receptor 4.

### 3.3 Peripheral coagulation parameters and PV hemostasis in relation to disease severity

To evaluate secondary hemostasis alongside primary hemostasis, coagulation parameters were correlated with liver disease severity, as indicated by the MELD score. As shown in [Supplementary Figure S1, significant correlations were observed for the APTT (*r* = 0.5574, *P* = .0008) and fibrinogen (*r* = −0.4707, *P* = .0115), while no significant trends were observed for thrombin time, factor XIII activity, or antithrombin III activity. Across vascular compartments (Figure 4)—including the SMV, PV, hepatic vein, and peripheral blood—no significant differences were found in INR (Figure 4A), APTT (Figure 4B), thrombin time (Figure 4C), factor XIII activity (Figure 4E), or antithrombin III activity (Figure 4F), suggesting a comparable hemostatic profile. However, fibrinogen levels (Figure 4D) were significantly lower in the PV compared with the hepatic vein (*P* = .0198).

**Figure 4:**
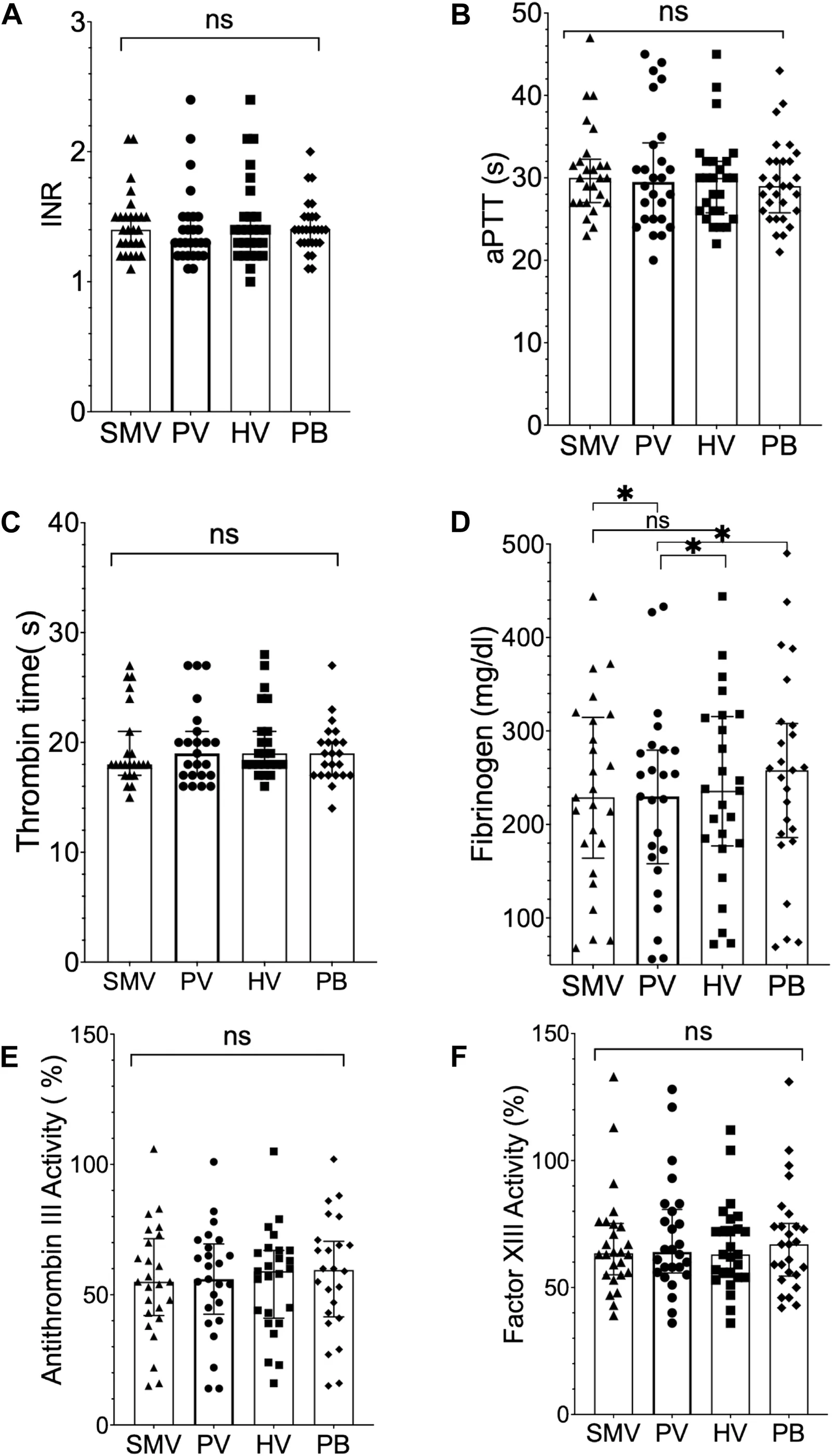
From the different coagulation parameters investigated, only portal venous fibrinogen is significantly reduced compared to superior mesenteric vein (SMV), liver vein, and PB. INR (A), activated partial thromboplastin time (APTT) (B), thrombin time (C), fibrinogen (D), factor XIII activity (E), and antithrombin III activity (F) were measured in the SMV, portal vein (PV), hepatic vein (HV), and peripheral venous blood (PB). Fibrinogen levels differed significantly across compartments (*P* = .0093), with lower levels in the portal vein compared with the HV (*P* = .0198), whereas no significant differences were observed for the other parameters (*n* = 20). Statistical analysis: Friedman test with Wilcoxon signed-rank test for post hoc comparisons. INR, international normalized ratio.

### 3.4 The impact of TIPS placement on platelet reactivity and count

To investigate the effects of TIPS placement on platelet activation and reactivity, both were assessed in PB before and 1 day after the procedure. Flow cytometric assessment revealed no significant changes in baseline expression of PAC-1 and CD62P, nor in ADP- or PAR4-induced platelet activation (Figure 5A–F). This indicates that TIPS placement does not cause acute alterations in platelet reactivity within this early timeframe.

**Figure 5:**
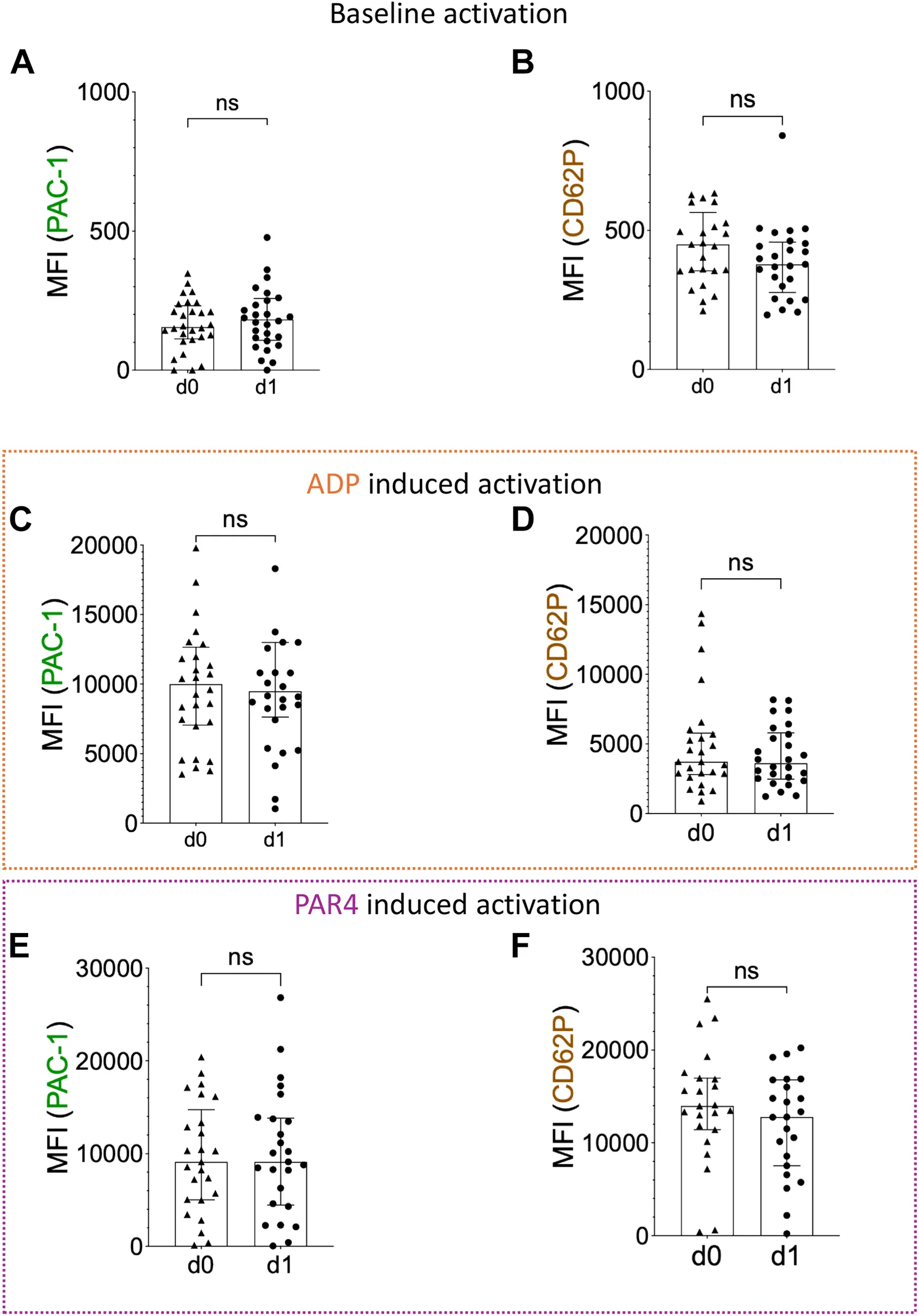
Transjugular intrahepatic portosystemic shunt placement does not affect basal and inducible platelet activation. Platelet activation was measured at baseline and 1 day after transjugular intrahepatic portosystemic shunt placement. No significant changes were observed in PAC-1 or CD62P expression at baseline (A, B), after adenosine diphosphate (ADP) stimulation (C, D), or after PAR4 stimulation (E, F) (*n* = 21-25). Statistical analysis: Wilcoxon signed-rank test. MFI, median fluorescence intensity; PAR4, protease-activated receptor 4.

To further investigate thrombocytopenia after TIPS, a larger cohort (*n* = 108) was examined. While no overall differences were initially detected (Figure 6A), platelet counts improved after TIPS in patients with advanced baseline thrombocytopenia (Figure 6B). In contrast, no significant change was observed in patients with baseline platelet counts above 100,000/μL (Figure 6C).

**Figure 6:**
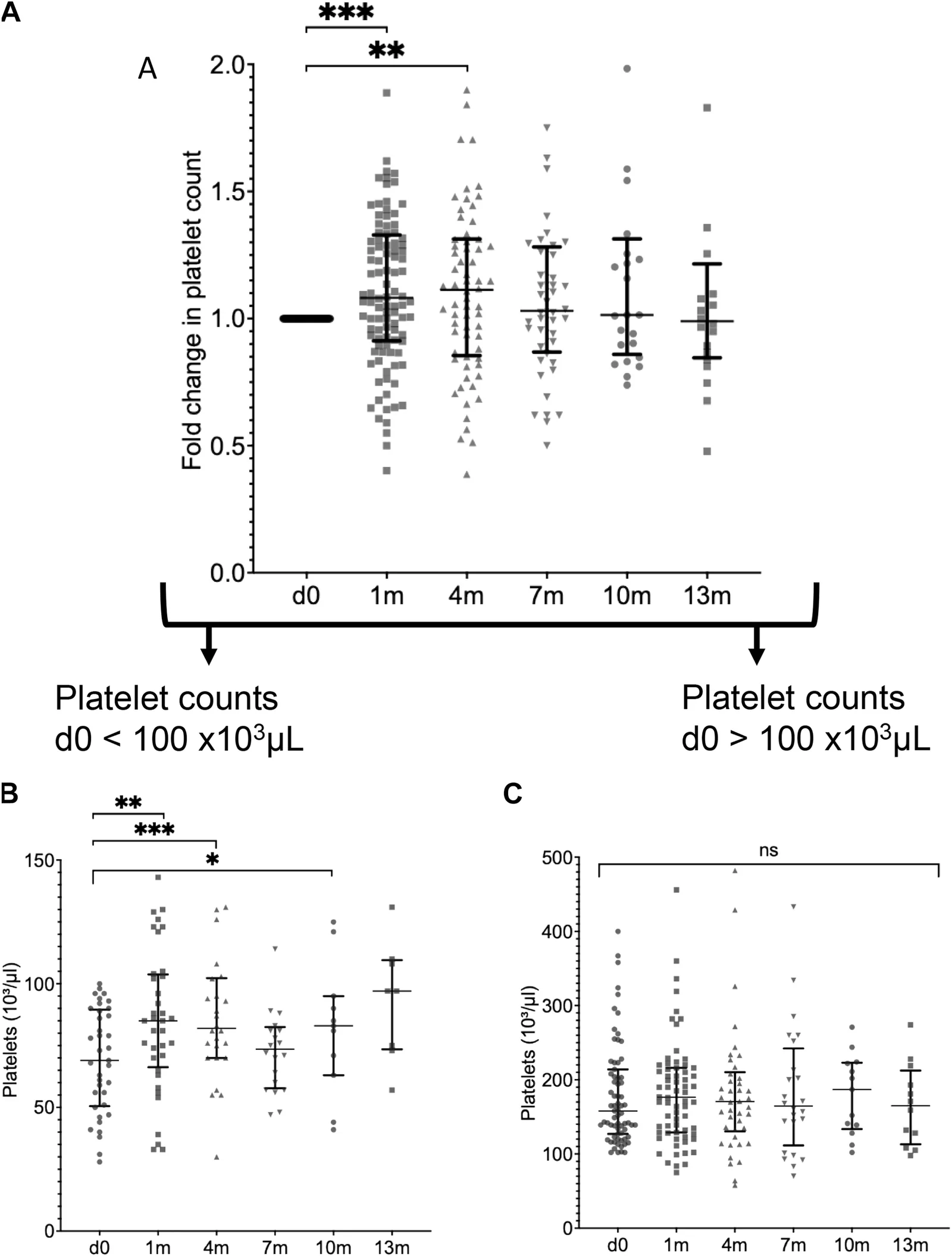
Longitudinal changes in platelet counts after transjugular intrahepatic portosystemic shunt (TIPS) are restricted to patients with baseline thrombocytopenia. (A) Fold change in platelet count over time relative to baseline (day 0) in the extended cohort (*n* = 108). A significant increase was observed at the first follow-up time point (1 month). (B) In patients with baseline thrombocytopenia (<100 × 10^3^/μL), absolute platelet counts increased significantly and remained higher than baseline for up to 10 months (*n* = 34). (C) Absolute platelet counts in patients with baseline values >100 × 10^3^/μL (*n* = 74). No significant changes were observed during follow-up. Statistical analysis: Friedman test with Dunn’s multiple comparison; *P* < .05.

### 3.5 Secondary hemostasis parameters remain stable 1 month after TIPS

Coagulation parameters reassessed 30 days after TIPS placement showed no significant changes in INR, APTT, thrombin time, fibrinogen, factor XIII activity, or antithrombin III activity compared with baseline (Supplementary Figure S2). These findings indicate that secondary hemostasis remains stable in the early postinterventional period.

## 4 Discussion

This study examined platelet counts, platelet activation, and coagulation profiles across different vascular compartments in patients with advanced cirrhosis undergoing TIPS placement. As expected, platelet counts were significantly reduced in patients with advanced disease, with the lowest values observed in those with severe cirrhosis. Platelet counts in the PV were significantly higher than in the hepatic vein, indicating that a substantial fraction of platelets remains in the liver as portal venous blood passes through it. However, flow cytometric analysis revealed no evidence of increased baseline platelet activation or enhanced responsiveness to ADP or PAR4 stimulation in any vascular compartment. Peripheral coagulation markers also correlated with disease severity, and fibrinogen levels were locally reduced in the PV. These findings suggest that, while platelet activation and reactivity and coagulation are altered in cirrhosis depending on disease severity, there is no clear evidence of localized platelet hyperactivation within the portal circulation.

End-stage liver disease is characterized by complex homeostatic disruption involving both primary and secondary components. Thrombocytopenia is a hallmark of cirrhosis and correlates with disease severity [33], a finding that is confirmed in our cohort (Figure 1A, B). Given the known association between thrombocytopenia and the development of PVT [5], patients with severe thrombocytopenia were not excluded, in contrast to previous studies [34].

In addition to thrombocytopenia, thrombocytopathy has also been reported in liver cirrhosis [[15], [35]]. In line with this, our data indicate that advanced liver disease is associated with enhanced PAR4-mediated platelet α-granule release, as reflected by increased CD62P surface expression (Figure 1C), in line with other studies indicating that patients with liver disease have increased CD62P expression [[35], [36], [37]]. It has been hypothesized [38] that increased platelet activation in patients with liver cirrhosis correlates with an increased risk of the formation of PVT.

In particular, endothelial injury [8] and the translocation of lipopolysaccharides (LPSs) resulting from impaired intestinal barrier function [[12], [39]] have been implicated in promoting local platelet activation and thrombus formation [[9], [10], [12]].

Consistent with this, von Willebrand factor, a key mediator of platelet adhesion, has been reported to correlate with disease severity [40] and be elevated in the portal circulation [11]. Against this background, the present study tested the hypothesis that platelet activation and reactivity would be increased in the PV, and to an even greater extent, in the SMV, where higher concentrations of proinflammatory mediators, such as LPSs, are expected [41]. This is particularly pertinent given that, according to the current literature, in patients with cirrhosis, increased translocation of bacterial components into the blood occurs due to a disrupted intestinal blood barrier. This aspect was initially elaborated in particular in the context of alcohol-related liver disease [42] and later also for other entities [[41], [43]].

Contrary to these expectations, our findings did not reveal increased basal platelet activation or enhanced responsiveness to PAR4 or ADP stimulation in portal or SMV blood compared with peripheral or hepatic venous blood. These findings differ from reports suggesting increased portal venous platelet activation or a prothrombotic portal venous milieu [[10], [11]]. However, previous studies assessed different components of hemostasis, including soluble Pselectin, soluble glycoprotein VI, von Willebrand factor, factor VIII, or endothelial injury markers. These markers may originate from platelets as well as nonplatelet sources, including endothelial cells [[14], [44]], and may be influenced by platelet count [45] and sample processing [[46], [47]]. Moreover, apparent gradients between portal and hepatic venous blood may at least partly reflect hepatic clearance of soluble activation markers [48]. Therefore, apparent portal-to-hepatic venous gradients in soluble markers do not necessarily indicate increased *in situ* platelet activation within the PV. Importantly, findings regarding such soluble markers are also inconsistent, as a recent study did not observe increased portal venous hemostatic, inflammatory, or endothelial activation compared with systemic blood [7]. In contrast, our study used flow cytometry–based assessment of platelet surface P-selectin expression and activated GPIIb/IIIa, allowing a more direct assessment of platelet activation at the single-platelet level [23].

The role of platelet activation in the development of PVT has been explored in 2 further reports [[17], [49]]. However, these studies only demonstrated that platelet activation, as measured in PB, is increased in cirrhotic patients with established PVT compared with those without thrombosis. These findings do not provide mechanistic insights into the pathophysiology underlying the onset of PVT.

Notably, the results summarized herein are consistent with those of a previous study [34], which also found no evidence of enhanced platelet activation in portal blood. However, that study only included patients with platelet counts greater than 100,000/μL. Similarly, another study [50] could not detect differences in platelet clotting function across compartments. Importantly, however, their study utilized rotational thromboelastometry, a technique influenced by platelet count [21]. The fact that significant intercompartmental differences in platelet numbers exist, as suggested herein (Figure 1D) and previously [9], underscores the need for platelet count-independent assessment techniques. By employing flow cytometry–based analysis of P-selectin expression and GPIIb/IIIa activation, this study provides a platelet count–independent evaluation of platelet activation and reactivity [[35], [38], [51]].

With regard to secondary hemostasis, our results confirm that alterations in pro- and anticoagulatory factors correlate with disease severity and thus with MELD score (Supplementary Figure S1). The observed lower fibrinogen concentration in the PV compared with the hepatic and peripheral veins may be due to increased local fibrin deposition and consumption or may reflect the hepatic origin of fibrinogen. Additionally, the influence of portal hypertension, which could cause dilution effects, and splenic sequestration may also contribute to this observation. These mechanisms remain speculative and warrant further investigation to clarify their exact role in cirrhotic patients with PVT.

Delahousse et al. [52] did not detect such differences in fibrinogen concentrations between portal and jugular venous samples. However, several studies suggest that none of the identified disorders of secondary hemostasis correlate with the development of PVT or can predict it reliably [[5], [6], [53]]. In addition, secondary hemostasis does not seem to be locally altered within the PV, as demonstrated by studies comparing portal and systemic coagulation profiles. However, this interpretation should be restricted to the coagulation parameters assessed in the present study. The absence of intercompartmental differences in conventional coagulation factors does not exclude local alterations in other thrombosis-related pathways, including thrombin generation, endothelial activation, contact system activation, intrinsic pathway activation, or immunothrombotic mechanisms. This is particularly relevant in light of recent data demonstrating activation of the contact system and intrinsic pathway in peripheral and portal venous circulations in patients with cirrhosis [54]. Furthermore, it should be noted that conventional plasma-based coagulation tests have limited sensitivity for detecting the rebalanced hemostatic state characteristic of cirrhosis and may therefore not fully capture subtle local prothrombotic alterations.

Overall, the observations presented herein support the hypothesis proposed by Turon et al. [5], who emphasized hemodynamic alterations—particularly congestion due to portal hypertension—as central to the pathogenesis of PVT. It should also be considered that cirrhosis is characterized by a rebalanced hemostatic system, comprising both pro- and anticoagulatory alterations affecting secondary hemostasis as well as platelet function. Notably, published data on platelet activation in cirrhosis are heterogeneous, with reports of both impaired platelet activation and reactivity and increased expression of activation markers, depending on the experimental approach and agonist used [[37], [55]].

Moreover, several studies have also discussed whether PVT is truly driven by enhanced thrombogenesis [[56], [57]].

In this context, TIPS placement could mitigate local conditions by improving portal blood flow. In this context, platelet activation and reactivity were correlated with PV velocity, but no increase in activation or reactivity was detected as portal flow decreased (Figure 3).

Despite the potential of TIPS to alter the thrombotic environment, no consensus exists regarding postprocedural anticoagulation or antiplatelet strategies [30]. The presence of biomaterials in the bloodstream can trigger undesired platelet activation, potentially leading to thrombotic complications [58]. It is therefore reasonable to question whether similar mechanisms apply to TIPS in order to develop uniform recommendations for anticoagulant therapy after TIPS placement [31].

In coronary interventions, platelet activation increases shortly after stent implantation [[59], [60]]. In contrast, our data did not demonstrate an acute rise in platelet activation 1 day after TIPS placement (Figure 5), possibly reflecting lower shear stress in the venous system. In contrast to the activation of platelets, the results corroborate previous observations [61] that TIPS implantation resulted in recovery from thrombocytopenia, particularly in patients with severe thrombocytopenia, which, in turn, may favor procoagulant mechanisms.

In summary, our findings do not indicate increased platelet activation or reactivity in the PV of patients with cirrhosis, as assessed by flow cytometry–based analysis of platelet P-selectin expression and GPIIb/IIIa activation. In addition, TIPS placement was not associated with acute platelet activation in the early postinterventional period. Within the limitations of our sample size and the selected hemostatic parameters assessed, these findings do not support the concept of pronounced PV-specific platelet hyperreactivity. Instead, they are compatible with the notion that hemodynamic alterations, including portal hypertension and venous stasis, may substantially contribute to PVT development. Larger prospective studies incorporating broader hemostatic profiling are warranted to further clarify the relative contribution of local hemostatic alterations and hemodynamic factors and to determine optimal antithrombotic strategies in cirrhotic patients undergoing TIPS.

One limitation of this study is the limited number of patients included in the flow cytometry analysis, which increases the risk of type II error and should therefore be considered when interpreting the absence of significant differences in platelet activation or reactivity. Accordingly, our negative findings cannot exclude smaller compartment-specific differences that may become detectable in larger cohorts. However, we did not observe consistent trends in basal platelet activation or agonist-induced platelet reactivity across vascular compartments that would suggest pronounced portal venous platelet hyperreactivity. It is also worth noting that comparable studies investigating platelet activation in similar contexts included similar or even smaller patient numbers [[10], [34], [62]]. For a more detailed assessment of TIPS-related platelet activation, future studies could also measure platelet activation locally at the level of the TIPS (eg, 1 day after implantation in the outflow of the stent), whereas our current approach captures only systemic activation. Notably, comparable studies investigating coronary stents have likewise assessed platelet activation systemically [[59], [60]]. Platelet activation could also be assessed at later time points, such as 1 week or 30 days post TIPS. In our department, patients are treated with clopidogrel and thus show reduced platelet activation at 30 days (data not shown). In centers where antiplatelet therapy is not routinely administered, similar measurements could be repeated to investigate the temporal dynamics of platelet activation.

A further limitation is that this study assessed platelet activation using surface markers only, alongside selected coagulation parameters. Therefore, other thrombosis-related pathways are not captured, including thrombin generation, extracellular vesicles, neutrophil activation, endothelial activation, contact system activation, or immunothrombosis. Broader hemostatic profiling will be required to more comprehensively characterize the portal venous thrombotic milieu in cirrhosis.

## Supplementary material

Supplementary Figure S1 Coagulation dysfunction correlates with stage of cirrhosis. (A) MELD score positively correlated with aPTT (*n* = 34; *r* = 0.5574, *P* = .0008). (B) MELD score showed no significant correlation with thrombin time (*n* = 29; *r* = 0.3452, *P* = .0779). (C) MELD score inversely correlated with fibrinogen levels (*n* = 28; *r* = –0.4707, *P* =.0115). (D) MELD score showed no significant correlation with Factor XIII activity (*n* = 29; *r* = –0.3305, *P* = .0922). (E) MELD score showed no significant correlation with antithrombin III activity (*n* = 28; *r* = –0.2126, *P* = 0.3077). Statistical analysis: Spearman correlation; linear regression lines shown for illustration. **Supplementary Figure S2** Coagulation Parameters Remain Unchanged 30 Days After TIPS. INR, aPTT, thrombin time, fibrinogen, Factor XIII activity, and antithrombin III activity were measured at baseline and day 30 after TIPS (*n* = 12–16). No significant changes were observed. Statistical analysis: Wilcoxon signed-rank test. **Supplementary Figure S3** Gating strategy for FACS analysis. Platelet populations were identified based on forward and side scatter (FSC/SSC) properties and previously defined by CD41 staining in preliminary experiments. A platelet gate was defined accordingly. Acquisition was set to terminate once 10,000 gated platelet events had been recorded.
