# Supplementary material for: Reevaluating hypercoagulability in the portal vein and the impact of transjugular intrahepatic portosystemic shunt on platelet activation in cirrhosis
Source: Res Pract Thromb Haemost. 2026 Sep 29;10(7):106977. doi: 10.1016/j.rpth.2026.106977 (PMC13696348; doi:10.1016/j.rpth.2026.106977)

Figure S1

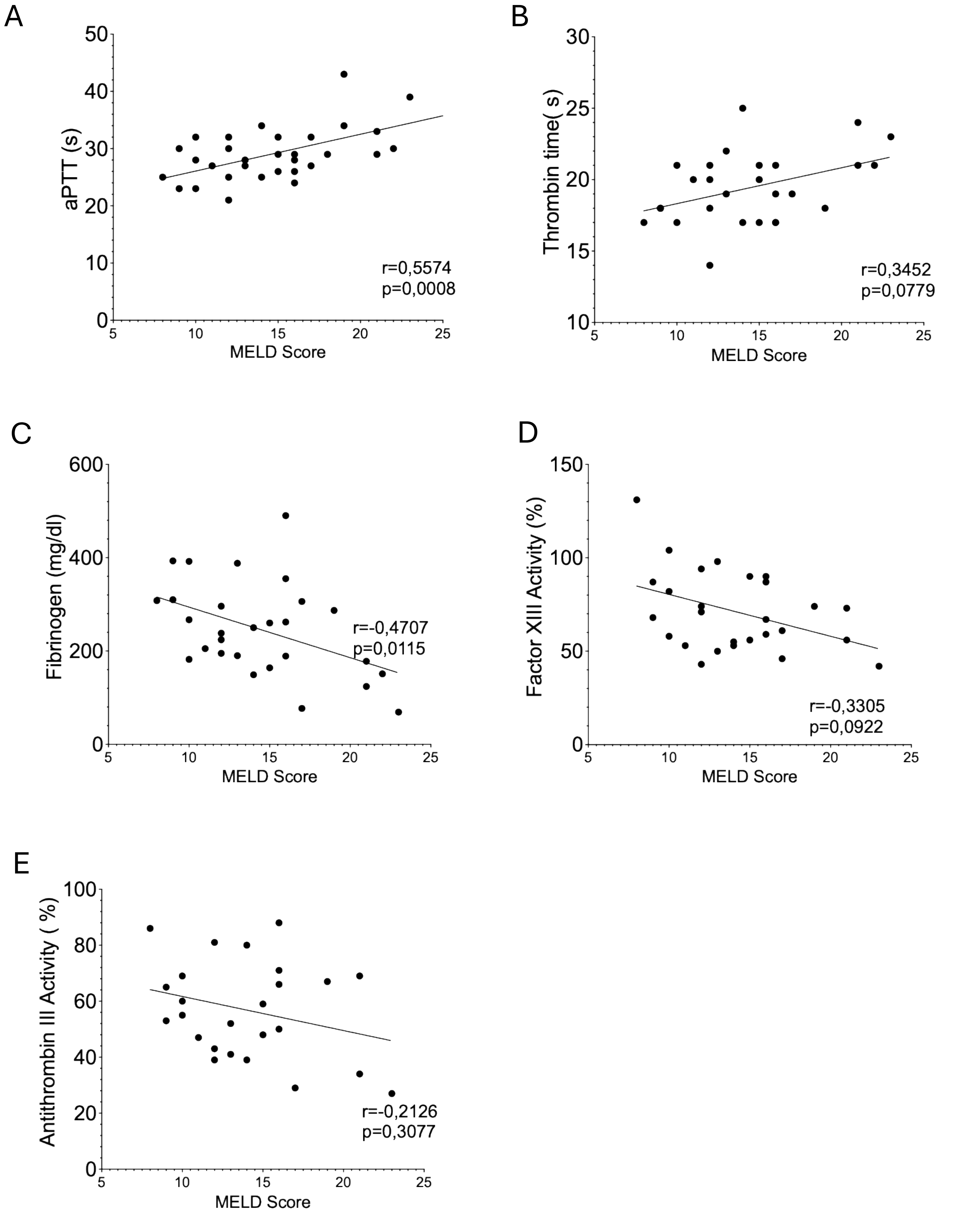
A
aPTT (s)
MELD Score
r=0,5574
p=0,0008
B
Thrombin time(s)
MELD Score
r=0,3452
p=0,0779
C
Fibrinogen (mg/dl)
MELD Score
r=-0,4707
p=0,0115
D
Factor XIII Activity (%)
MELD Score
r=-0,3305
p=0,0922
E
Antithrombin III Activity (%)
MELD Score
r=-0,2126
p=0,3077

Figure S2

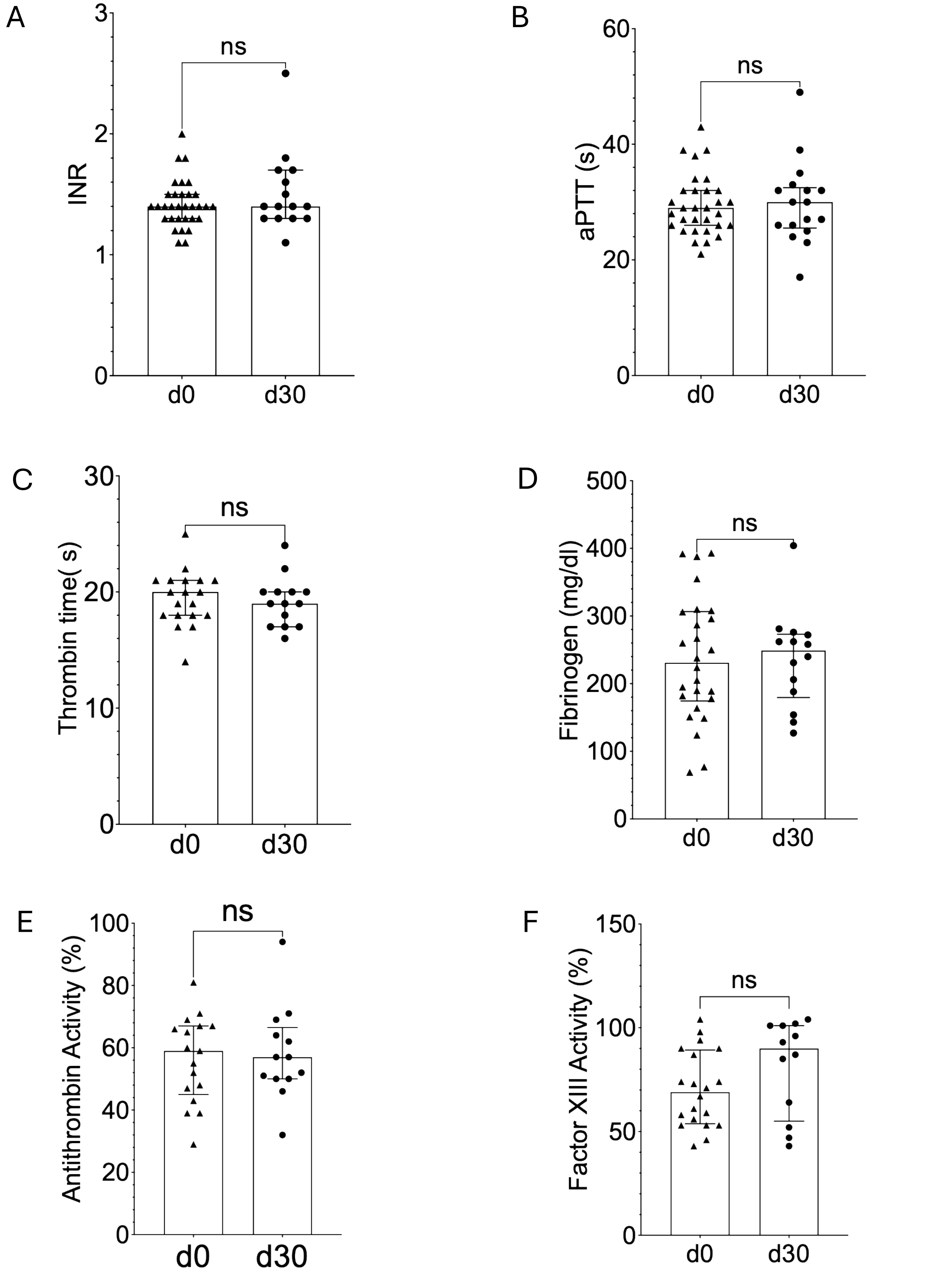
A
INR
ns
d0
d30
B
aPTT (s)
ns
d0
d30
C
Thrombin time(s)
ns
d0
d30
D
Fibrinogen (mg/dl)
ns
d0
d30
E
Antithrombin Activity (%)
ns
d0
d30
F
Factor XIII Activity (%)
ns
d0
d30

Figure S3

A: Gating of the platelet population based on FSC and SSC

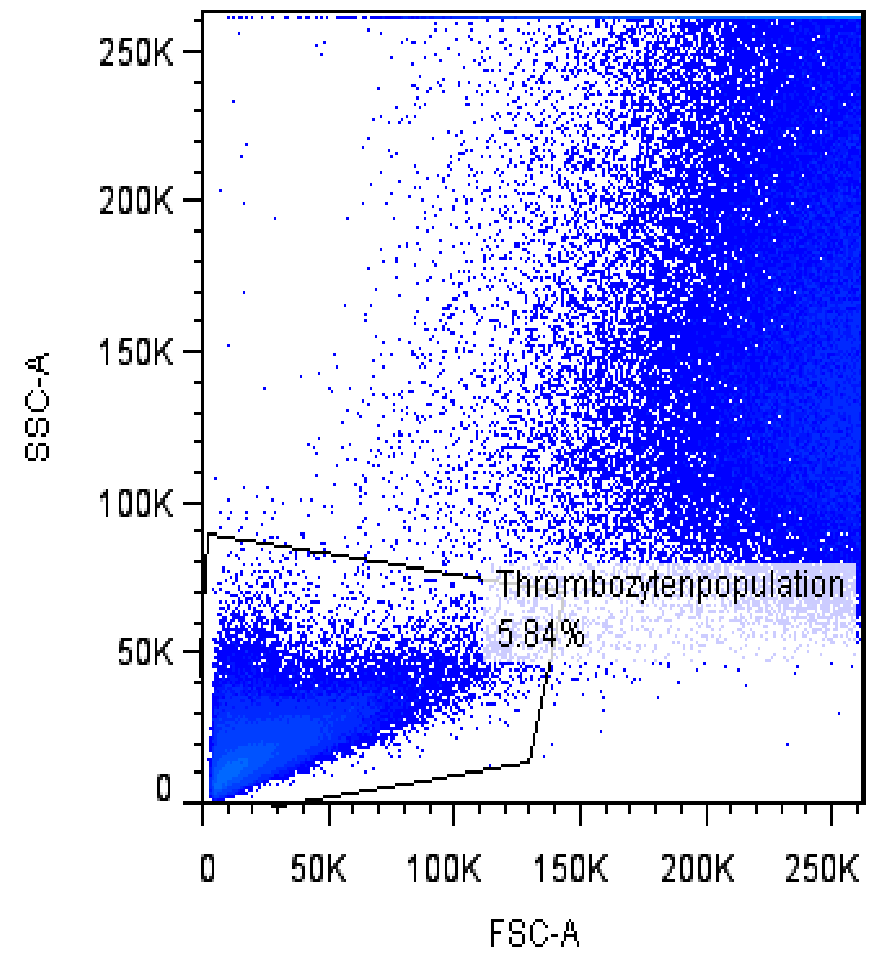

B: Background fluorescence of the unstained control sample (Pac-1)

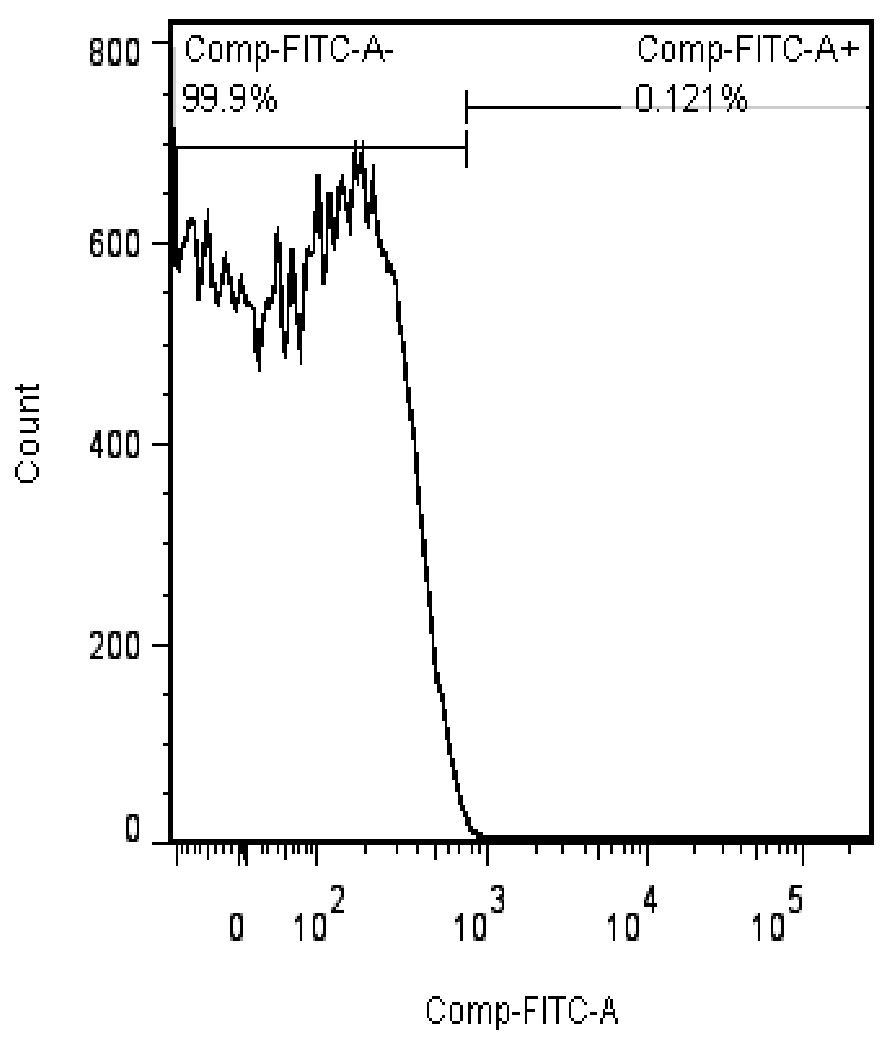

C: Definition oft the PAC-1 positive population

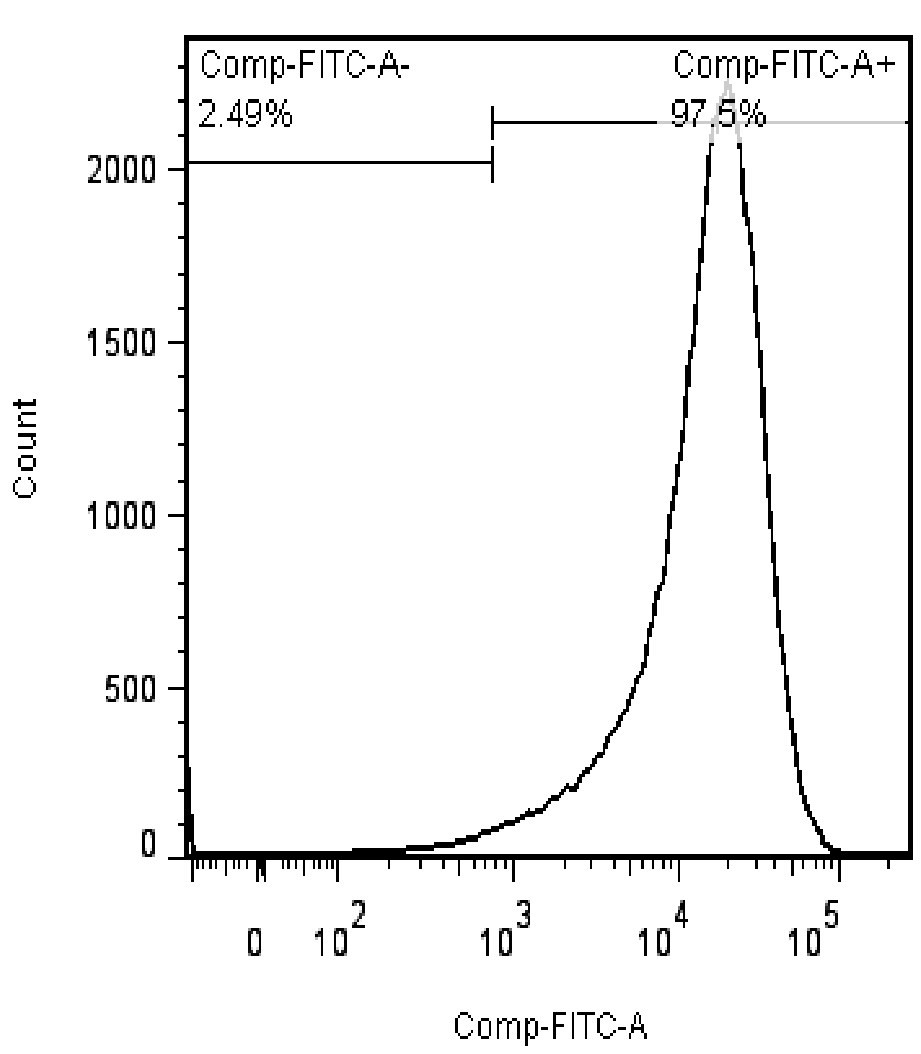

D: Background fluorescence of the unstained control sample (CD62P)

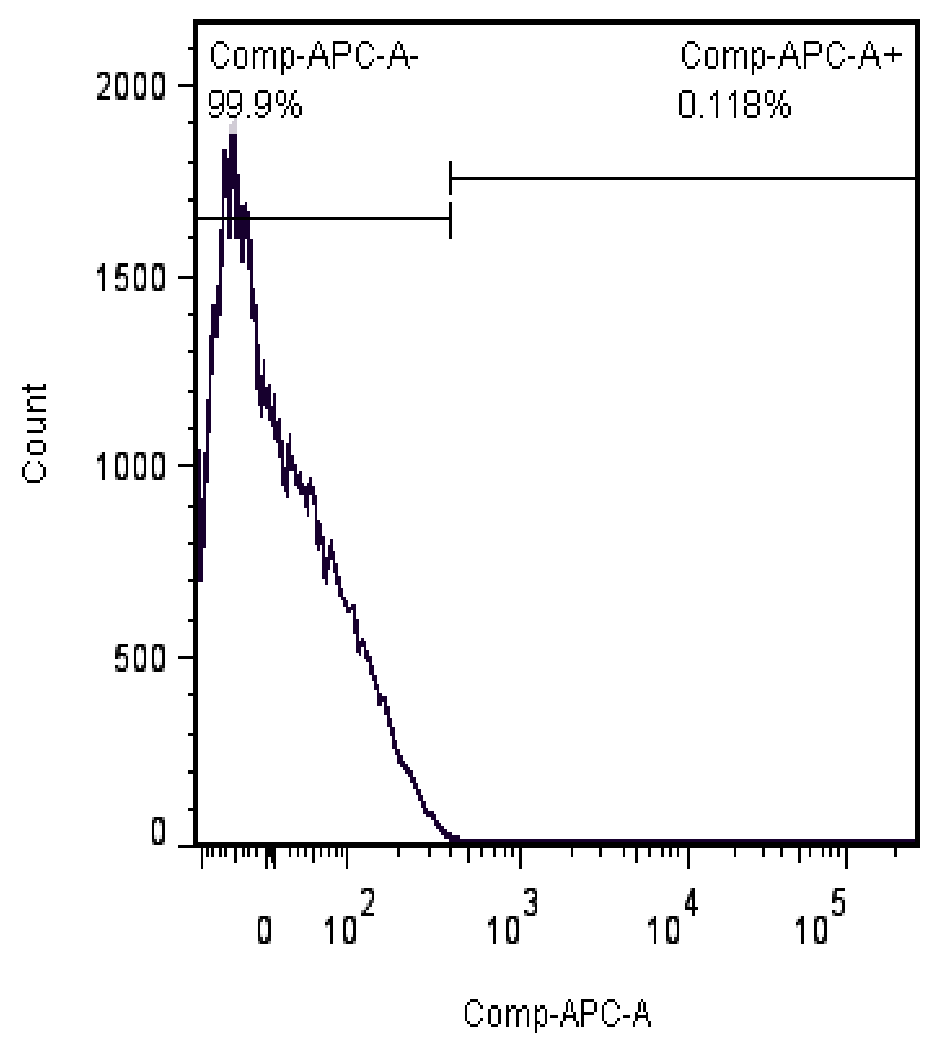

E: Definition oft the CD62P positive population

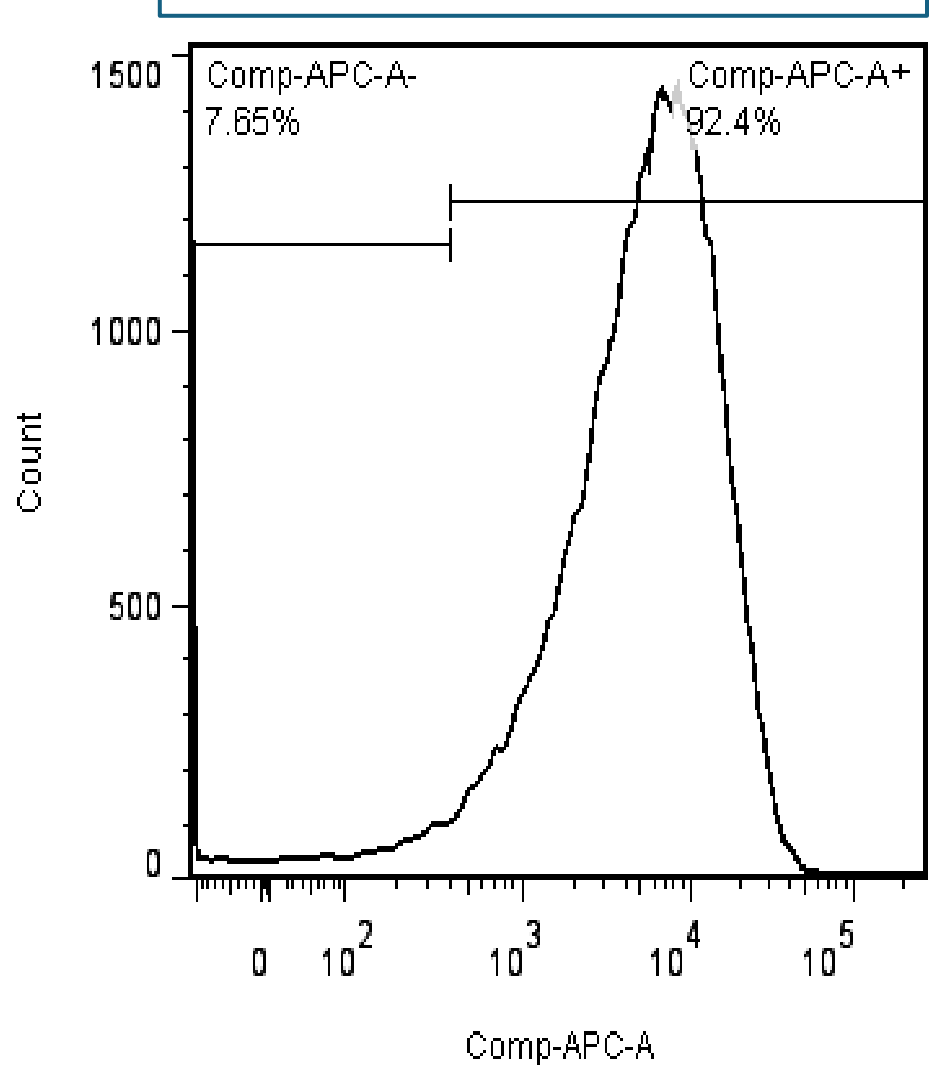

Supplement: Supplementary file 1 [file mmc1.pdf]
